# 42. Consumption of Swietenia macrophylla seeds can lead to hepatitis and autoimmune phenomena

**DOI:** 10.1093/rap/rky034.005

**Published:** 2018-09-20

**Authors:** Joanna, Wai Yee Leong, Wint Wah Soe, Archana Vasudevan, Eng Kiong Teo, Kwong Ming Fock

**Affiliations:** Medicine, Changi General Hospital, Singapore, SINGAPORE

**Introduction:** Medication, herbal and dietary supplement induced lupus and liver injuries have been well documented in the literature. We report two cases of skyfruit seed induced autoimmune phenomena and hepatic injury. Swietenia macrophylla seeds, also known as skyfruits or mahogany seeds in Southeast Asia have been used as a remedy for diabetes, hyperlipidemia and shown to have antibiotic properties by its effect on the innate immune system. Due to its perceived benefits, it is being used as part of traditional medication in Southeast Asia for various ailments.

**Case description:** A 62 year-old Indian male with a history of diabetes mellitus, hypertension, hyperlipidaemia, coronary artery disease and non-alcoholic fatty liver disease presented to the gastroenterology clinic for transaminitis. He had been on stable doses of medications for the above including atorvastatin 10mg ON for the last one year. He presented with polyarthralgia with tenosynovitis and severe restriction of motion of his right shoulder, left wrist and dorsum of right hand at onset with minimal response to a short course of prednisolone. His symptoms improved but were still present until review in the rheumatology clinic six weeks later. Of note he had taken skyfruit seeds six weeks ago. Systemic review was negative for rashes, oral ulcers, sicca symptoms, Raynaud's, hematuria, frothy urine or abdominal pain. He reported a significant unintentional weight loss of 14kg in the preceding 12 months. There was no family history of autoimmune conditions. Physical examination showed synovitis of the right metacarpophalangeal and proximal interphalangeal joints. Full blood count, renal function and thyroid function tests were normal. His liver function test showed transaminitis with alanine transaminase (ALT) of 699 U/L , aspartate aminotransferase (AST) of 645 U/L and gamma-glutamyl transferase (GGT) of 125 U/L. C-reactive protein (CRP) was 75.7 mg/L. Anti-nuclear antibody (ANA) screen was positive; anti-ENA (extractable nuclear antigen) profile and rheumatoid factor were negative. Anti-mitochondrial M2, anti- liver cytosol 1, anti- liver kidney microsomal and anti- soluble liver antibodies were negative. Hepatitis B surface antigen and antibodies against hepatitis A, C and E were non-reactive. He realized that the onset of symptoms - arthritis, weight loss and transminitis correlated with his intake of skyfruit seeds and stopped consuming them. The inflammatory arthritis had completely resolved and CRP and liver enzymes had markedly improved six weeks later. Within a year, his liver function tests had normalised and he had no recurrence of his arthritis.

A 59 year-old Chinese female, previously well, presented with right upper quadrant discomfort for two weeks. Liver function tests were deranged with a total bilirubin 1.51 umol/L, ALT 859 U/L, AST 491 U/L and GGT 98 U/L. Hepatitis B, hepatitis C and human immunodeficiency virus (HIV) antibodies were non-reactive. A CT (Computed tomography scan) of the abdomen and pelvis showed fatty liver, and mesenteric panniculitis with prominent mesenteric nodules. She had noted a 6kg weight loss over six weeks and loss of appetite. Oesophagogastroduodenoscopy and colonoscopy showed chronic atrophic gastritis without dysplasia or malignancy. She had started taking five sky fruit seeds daily for duration of one month prior to presentation. Systemic review was negative for features of connective tissue diseases. Her physical examination was normal except for dry skin over hands and feet related to frequent ablutions. Her transaminitis resolved one month after stopping consumption of the seeds. Laboratory testing for autoimmune conditions showed a positive ANA 1:320, negative anti-double stranded deoxyribonucleic acid (DNA), anti-ENA profile and anti-neutrophil cytoplasmic antibody (ANCA). She had an elevated aldolase and creatinine kinase (CK) at 30.8 U/L and 463 U/L respectively. These normalised two weeks after stopping her recently initiated exercise regimen. Further work-up showed normal full blood count, renal function, thyroid function tests, erythrocyte sedimentation rate, needle electromyography of proximal and distal limbs and high resolution CT thorax.

**Discussion:** To our knowledge, these are the first reports of Swietenia macrophylla seeds’ association with liver injury and induction of autoimmune phenomena such as inflammatory arthritis, mesenteric panniculitis and positive ANA. Our patients did well on prompt discontinuation of the skyfruit seed intake. These reports emphasize the importance of being aware of the regional trend in herbal supplement intake as there has been an increase in advertising by local markets about the benefits of skyfruit seeds. This may lead to serious health sequelae if not recognized promptly and discontinued.

**Key Learning Points:** Consumption of Swietenia macrophylla seeds can lead to hepatitis and autoimmune phenomena.

**Disclosure: J. Leong:** None. **W. Soe:** None. **A. Vasudevan:** None. **E. Teo:** None. **K. Fock:** None.

